# Cerebral Autoregulation Is Disrupted Following a Season of Contact Sports Participation

**DOI:** 10.3389/fneur.2018.00868

**Published:** 2018-10-22

**Authors:** Alexander D. Wright, Jonathan D. Smirl, Kelsey Bryk, Sarah Fraser, Michael Jakovac, Paul van Donkelaar

**Affiliations:** ^1^MD/PhD Program, University of British Columbia, Vancouver, BC, Canada; ^2^Southern Medical Program, Reichwald Health Sciences Centre, University of British Columbia Okanagan, Kelowna, BC, Canada; ^3^Experimental Medicine Program, Faculty of Medicine, University of British Columbia, Vancouver, BC, Canada; ^4^School of Health and Exercise Sciences, University of British Columbia, Kelowna, BC, Canada; ^5^Department of Kinesiology and Applied Physiology, University of Delaware, Newark, DE, United States

**Keywords:** cerebral blood flow, autoregulation, blood pressure, transfer function analysis, repetitive subconcussive head impacts

## Abstract

Repetitive subconcussive head impacts across a season of contact sports participation are associated with a number of deficits in brain function. To date, no research has investigated the effect of such head impact exposure on dynamic cerebral autoregulation (dCA). To address this issue, 179 elite, junior-level (age 19.6 ± 1.5 years) contact sport (ice hockey, American football) athletes were recruited for pre-season testing. Fifty-two non-concussed athletes returned for post-season testing. Fifteen non-contact sport athletes (age 20.4 ± 2.2) also completed pre- and postseason testing. dCA was assessed via recordings of beat-by-beat mean arterial pressure (MAP) and middle cerebral artery blood velocity (MCAv) using finger photoplethysmography and transcranial Doppler ultrasound, respectively, during repetitive squat-stand maneuvers at 0.05 and 0.10 Hz. Transfer function analysis was used to determine *Coherence* (correlation), *Gain* (response amplitude), and *Phase* (response latency) of the MAP-MCAv relationship. Results showed that in contact sport athletes, *Phase* was reduced (*p* = 0.027) and *Gain* increased (*p* < 0.001) at post-season compared to pre-season during the 0.10 Hz squat-stand maneuvers, indicating cerebral autoregulatory impairment in both the latency and magnitude of the response. Changes in *Phase* were greater in athletes experiencing higher numbers and severity of head impacts. By contrast, no changes in dCA were observed in non-contact sport controls. Taken together, these results demonstrate that repetitive subconcussive head impacts occurring across a season of contact sports participation are associated with exposure-dependent impairments in the cerebrovascular pressure-buffering system capacity. It is unknown how long these deficits persist or if they accumulate year-over-year.

## Introduction

Sport-related concussion is a global public health issue, with growing concern over the effects of repetitive subconcussive head impacts (1). Subconcussion can be defined as head impact that does not elicit signs or symptoms typical of concussion (2). A host of studies have revealed this type of exposure—experienced during participation in sports in which head impacts are common—is associated with various deficits to brain structure and function at a subclinical level: transient blood-brain barrier damage (3), alterations in white matter microstructure (4–6), altered cerebrovascular sensitivity to carbon dioxide (7), disrupted cerebral metabolism (8), altered resting functional connectivity (9), and altered task-based cortical activation patterns (10). Many of these studies used head impact sensors whose accuracy has been questioned (11–14) and some did not include non-contact sport control athletes thus potentially limiting the interpretation of their results. Despite these limitations, data from studies such as these has led to growing concern of the potential for such deficits to accumulate across multiple years of exposure to the development of irreversible changes that are accompanied by deterioration in clinical function (15). To further elucidate this possibility, it is necessary to first identify which systems appear to be most susceptible to repetitive subconcussive head impacts.

We have recently demonstrated using transcranial Doppler ultrasound that acute concussion leads to alterations in cerebral blood flow (CBF) as it pertains to neurovascular coupling (NVC) dynamics (16). By contrast, alterations in NVC dynamics are not apparent after a season of subconcussive head impacts (17). Multiple controllers of CBF exist beyond NVC dynamics including reactivity to carbon dioxide (CO_2_) and the blood pressure (BP) buffering system (18). With respect to the latter, in the face of changing BP, CBF is maintained via alterations to resistance within the cerebrovascular tree. The ability of the cerebrovasculature to buffer rapid changes in BP—referred to as dynamic cerebral autoregulation (dCA)(19)—is an important marker of cerebrovascular function, and encompasses myogenic, neurogenic, and metabolic mechanisms (20). Our group has recently demonstrated that dCA is systematically altered following acute concussion (21) and an outstanding question is whether exposure to repetitive subconcussive head impacts is associated with alterations in dCA. Such acute and chronic alterations would be consistent with the hypothesis that disruptions to dCA underlie persistent post-concussion symptoms (22, 23). Accordingly, our objective was to prospectively evaluate the effect of repetitive subconcussive head impact exposure on dCA indices, with deficits hypothesized to be observed at post-season relative to pre-season in a group of young adult elite contact sport athletes.

## Materials and methods

### Study design

One hundred and seventy-nine elite male (mean age 19.6 ±1.5 years) junior hockey (*n* = 90) and football (*n* = 89) athletes as well as 15 non-contact sport controls (mean age 20.4 ± 2.2 years; 12 cross-country running, 1 ultimate frisbee, 2 basketball) were recruited to the study. They underwent baseline laboratory testing prior to the beginning of the athletic season (preseason). Testing was repeated within 2 weeks of the end-of-season (post-season) for all non-contact sport controls and a subset of contact sport participants (*n* = 52). An additional 109 contact sport participants did not complete post-season testing for one of the following reasons: (i) traded to a team in a different city, (ii) returned to their hometown immediately following the end of the season, (iii) unable to attend a testing session within 2-weeks of season's end, or (iv) injury preventing the completion of testing. The remaining 18 contact sport athletes were diagnosed by team physicians and medical staff with a concussion during the season based on criteria outlined in the 4th Consensus Statement (24) and followed a different post-injury protocol that has been reported in a recent publication (21). Although the number of control subjects is relatively low, the differences we observed in the current study are larger than the within-subject coefficients of variation for dCA measures (10–15%) reported for healthy participants by our group (25). Thus, we are confident the sample size of the control group did not impact the interpretation of the current findings.

At the beginning of each testing session, participants completed the Sport Concussion Assessment Tool, version 3 (SCAT3) (24) including a graded symptom checklist (7-point Likert scale for 22 concussion symptoms), the Standardized Assessment of Concussion (SAC), as well as the modified Balance Error Scoring System (BESS). No participants were excluded based on predefined criteria including a significant history of cardiorespiratory, cerebrovascular, neurological, or severe neurodevelopmental disorder. All subjects underwent familiarization of testing procedures, and were asked to abstain from exercise, caffeine, and alcoholic beverages for 12^+^ h prior to testing. The experiment was performed in accordance with the ethical standard as laid down in the 1964 Declaration of Helsinki. Written informed consent was obtained prior to participation and the study protocol was approved by the Clinical Research Ethics Board at the University of British Columbia.

### Instrumentation

A three-lead electrocardiogram (ECG) recorded R-R intervals and heart rate. Cerebral blood velocity was recorded unilaterally in the middle cerebral artery (MCAv) using transcranial Doppler ultrasound (ST3, Spencer Technologies, Seattle, WA, United States). Once vessels were identified and signals optimized according to depth, waveform, and velocity (25), the ultrasound probes were locked in place with a fitted headframe. Beat-to-beat BP was continuously monitored with finger photoplethysmography (Finometer PRO, Finapres Medical Systems, Amsterdam, Netherlands) (26), while partial pressure of expired CO_2_ (P_ET_CO_2_) was sampled using an online gas analyzer (ML206, AD Instruments, Colorado Springs, CO, United States). All data were recorded at 1,000 Hz (PowerLab 8/30 ML880, AD Instruments) and stored for offline analysis using commercially available software (LabChart version 7.1, AD Instruments).

### Experimental protocol

All testing occurred at the same time of day and involved a hemodynamic challenge protocol. In particular, repetitive squat-stand maneuvers were used to induce rapid and systematic oscillations in BP (25). First, spontaneous fluctuations in physiological metrics were recorded while standing for 5 min. During squat-stand maneuvers, participants began in a standing position, squatted to hold a knee angle of ~90°, then returned to standing. Squat-stands were performed for 5 min at each of two metronome-paced frequencies, 0.05 and 0.10 Hz (i.e., 1 cycle every 20 or 10 s, respectively)–thought to reflect myogenic and autonomic contributions toward dCA, respectively (27). This active technique was chosen for the current study because it has been shown to result in markedly better between-day reproducibility relative to spontaneous or passive (oscillatory lower-body negative pressure) maneuvers (27).

### Data processing

**Figure 1:**
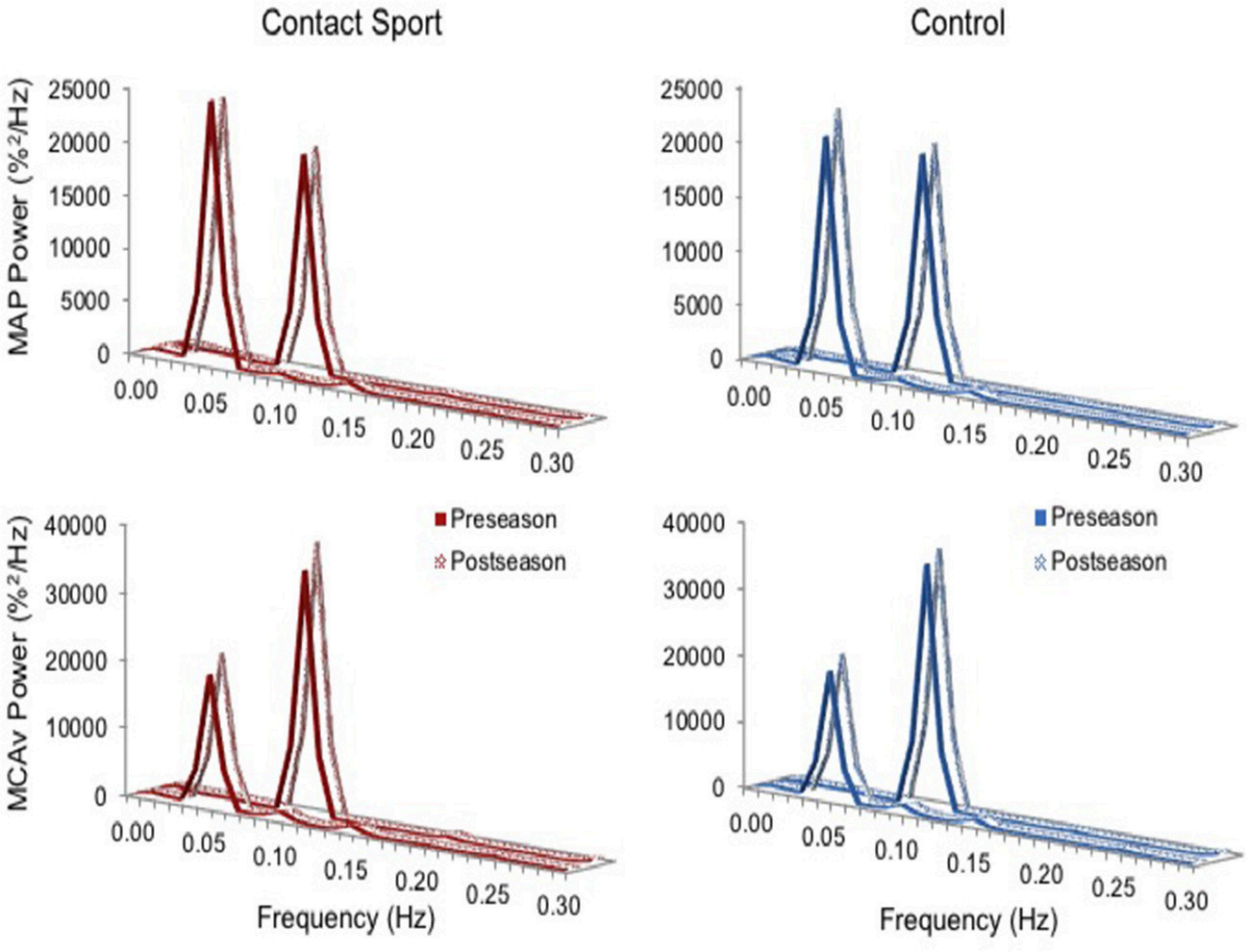
Normalized power spectrum densities for mean arterial pressure (MAP, **top**) and middle cerebral artery blood velocity (MCAv, **bottom**) for preseason and post-season squat-stands in contact sport athletes (red, **left**) and in non-contact sport control athletes (blue, **right**). The frequency at which PSD reached peak amplitude (either 0.05 or 0.10 Hz) was used for sampling point estimates for *Coherence, Phase*, and *Gain*.

Real time, beat-to-beat mean values of arterial pressure (MAP) and MCAv were determined from each R-R interval. All data were processed and analyzed with custom-designed software in LabView 14 (National Instruments, Austin, TX, United States). In accordance with recently published best-practice guidelines, the CARNet algorithm was used to complete the transfer function analysis (27). Figure 1 highlights the resulting augmented signal-to-noise ratios at the frequencies associated with the squat-stand maneuvers (0.05 or 0.10 Hz) providing the rationale for sampling *Coherence, Phase*, and *Gain* at these frequencies, falling within the very low frequency (VLF; 0.02–0.07 Hz) and low frequency (LF; 0.07–0.20 Hz) ranges where dCA is operant (28). *Coherence* characterizes the proportion of variance in MCA*v* explained by MAP; high coherence improves reliability of *Phase* and *Gain* estimates (25). *Phase* measures the timing offset between MAP and MCA*v* oscillations, whereas *Gain* provides a ratio of MCA*v* amplitude to MAP amplitude. With intact dCA, high *Phase* indicates rapid adjustment of cerebrovascular resistance to changing MAP, whereas low gain indicates low magnitudes of MAP oscillation transferred to the cerebrovasculature (i.e., greater buffering) (26). Phase wraparound was not present for any point-estimates.

### Head impact monitoring

During games throughout the season, a subset of players who completed both pre- and post-season testing wore impact sensors (xPatch, X2 Biosystems, Seattle, WA, United States) on the right mastoid (*n* = 29) to estimate linear and rotational accelerations during each head impact. Acceleration profiles were recorded for 100 ms (10 ms pre-impact, 90 ms post-impact) at 1,000 Hz when translational acceleration exceeded a 10 *g* threshold. Following each game, data were uploaded using the Head Impact Monitoring System (X2 Biosystems). Features within the software provide estimates of peak linear (PLA) and peak rotational (PRA) acceleration for each detected impact. Pilot testing of the devices during preseason non-contact practices revealed multiple impact events being recorded within the 10–20 *g* range during regular non-contact events such as hard stops or cuts. Previous work has demonstrated marked reduction in false-positive impacts when a 20 vs. 10 *g* threshold was used (29). Only acceleration events exceeding a 20 *g* threshold were used in subsequent analyses (30). Cumulative exposure to linear (*cPLA*) and rotational (*cPRA*) acceleration were estimated by summing across all impacts for the season. Non-contact sport athletes were not monitored for head acceleration events during their competitive season.

### Statistical analysis

Effects of exposure to repetitive subconcussive head impacts during the course of a competitive season were estimated using a 2 (group: contact vs. non-contact) × 2 (time: preseason vs. post-season) × 2 (frequency: 0.05 vs. 0.10 Hz) three-way mixed-ANOVA. Secondary exploratory analyses were conducted using independent samples *t*-tests to explore differences in change scores from pre- to post-season for Phase (ΔPhase) and Gain (ΔGain) between high and low quartiles for estimated impact exposure variables (Hits/season, cPLA, cPRA). Spearman's correlation coefficients were calculated between ΔPhase/ΔGain and change in SCAT3 scores from pre- to post-season. All statistical analyses were performed using SPSS version 22.0 for Macintosh (IBM Corp., Armonk, NY, United States). Shapiro-Wilks tests were used to assess for normality.

Bonferroni correction was used to correct for multiple comparisons to achieve a family-wise error rate of 0.05.

## Results

### Participant demographics

Demographic characteristics, SCAT3 scores, and resting physiological data are outlined in Table 1. No differences in dCA function were observed at preseason between participants who did vs. did not complete follow-up testing or between those participants who did or did not wear the impact sensors throughout the season. In addition, no differences in head impact exposure were apparent in those participants who completed the study vs. those who were lost to follow up. Biomechanical descriptors of impact exposure for participants who completed both the pre- and post-season testing are presented in Table 2. Relative to football players, ice hockey players experienced fewer hits per game, but greater cumulative number of hits due to season length differences (62 games in hockey vs. 10 games in football).

**Table 1:** Demographics, SCAT3 performance, and resting physiological parameters for contact and non-contact sport (Control) athletes at preseason and post-season.

**Metric**	**Contact sport (*****n*** = **52)**		**Control (*****n*** = **15)**	
	**Preseason**	**Post-season**	**Preseason**	**Post-season**
Age (years)	19.6 (1.5)		20.4 (2.2)	
BMI (kg/m^2^)	28.2 (4.9)		22.6 (3.0)	
Test Interval (days)	109.2 (25.8)		100.1 (23.8)	
# of Symptoms^a^	3.7 (3.6)	5.4 (5.0)	5.5 (5.0)	5.7 (3.3)
Symptom Severity^a^	6.7 (7.8)	9.6 (10.1)	8.1 (8.3)	8.2 (7.2)
SAC Score^a^	26.6 (1.9)	26.6 (1.8)	27.7 (1.5)	27.9 (1.4)
BESS Score^a^	3.7 (3.3)	2.9 (3.2)	2.7 (2.4)	2.6 (3.6)
MAP (mmHg)	92.2 (12.2)	92.6 (12.7)	93.9 (13.8)	91.3 (9.7)
MCAv (cm/s)	54.5 (9.3)	53.8 (7.5)	55.6 (14.4)	57.3 (14.3)
HR (bpm)	74.5 (10.0)	78.3 (11.9)	71.0 (9.9)	72.8 (13.8)
P_ET_CO_2_ (mmHg)	38.0 (3.0)	37.1 (2.6)	37.9 (1.8)	37.9 (1.3)

a Components of the Sport Concussion Assessment Tool, version 3.

*SAC, Standardized Assessment of Concussion; BESS, Balance Error Scoring System; MAP, mean arterial pressure; MCAv, middle cerebral artery velocity; HR, heart rate; P_ET_CO_2_, end-tidal partial pressure of carbon dioxide*.

**Table 2:** Descriptives (median, IQ range) for head impact exposure across subset of hockey (*n* = 10) and football (*n* = 19) players wearing impact sensors during the season.

**Metric**	**Hockey**	**Football**	***p***
Hits/game (#)	8.2 (6.1–11.2)	16.6 (8.7–21)	0.003
Hits/season (#)	353 (29–587)	166 (63–212)	0.002
PLA/hit (*g*)	36.3 (35–37)	36.6 (34–40)	0.448
cPLA (*g*)	11,920 (10,788–21,570)	5,794 (2,507–7,117)	0.002
PRA/hit (rad/s^2^)	5,036 (4,772–5,510)	6,601 (6,104–7,441)	< 0.001
cPRA (rad/s^2^)	2,016,603 (11,592–24,322)	1,057,691 (486,976–1,376,735)	0.003

p-values reflect results of Mann-Whitney U-tests comparing across sport.

*PLA, peak linear acceleration; PRA, peak rotational acceleration; cPLA/cPRA, cumulative PL*.

### Dynamic cerebral autoregulation

**Figure 2:**
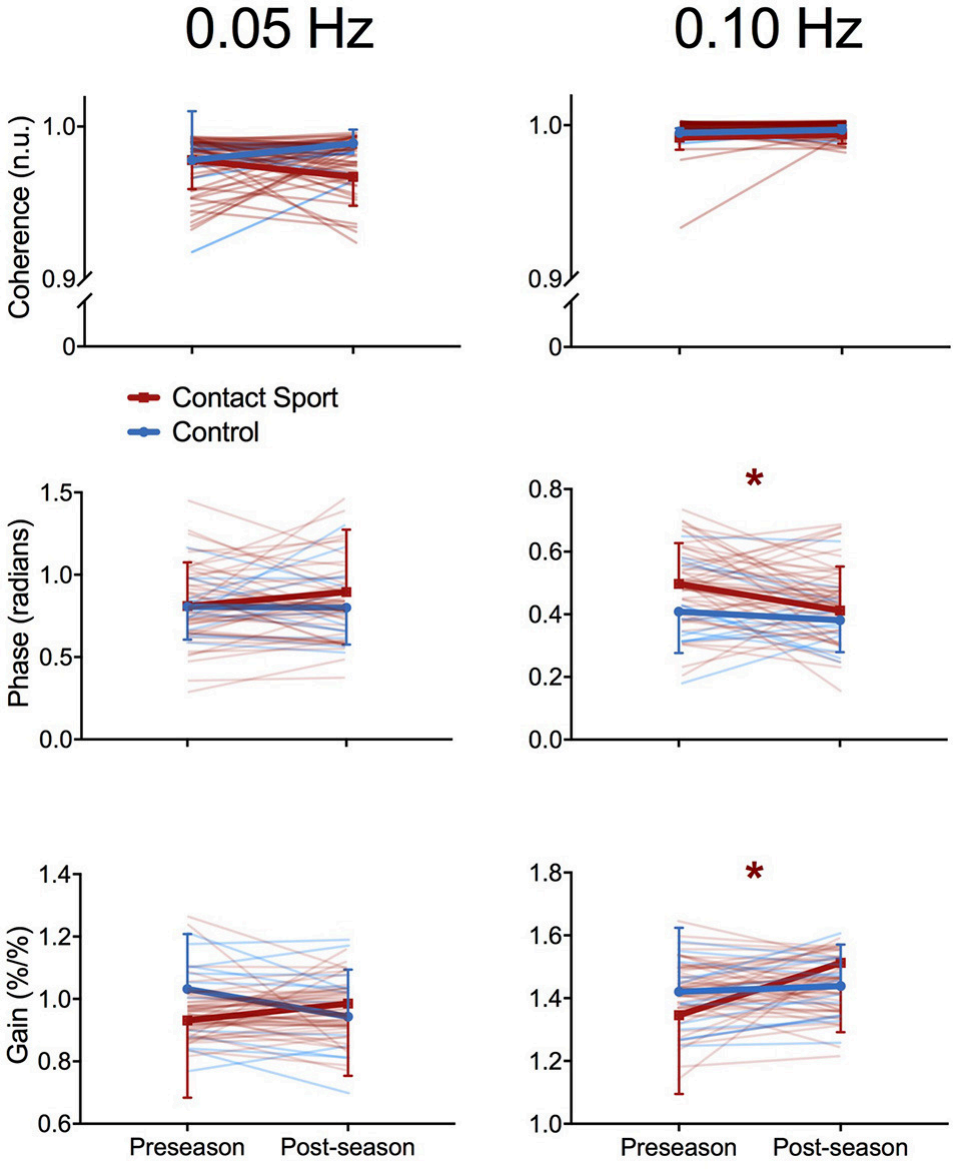
Transfer function analysis outcomes for each individual subject (pale lines) and for group averages (bold lines) for *Coherence* **(top)**, *Phase* offset **(middle)**, and *Gain* (bottom) during squat-stand maneuvers performed at 0.05 Hz **(left)** and 0.10 Hz **(right)**, assessed at preseason and postseason in contact sport (red lines) and non-contact sport control (blue lines) athletes. ^*^indicates significant simple effects of time in the contact sport group; no significant differences were observed in non-contact sport athletes. Error bars, standard deviation.

As expected, the squat-stand maneuvers substantially increased the power spectra of MCAv and MAP signals at the target frequencies (Figure 1). The three-way interaction term (frequency^*^time^*^group) was non-significant for *Coherence* [*F*_(1, 65)_ = 2.415, *p* = 0.125], *Phase* [*F*_(1, 65)_ = 1.494, *p* = 0.226], and *Gain* [*F*_(1, 65)_ = 0.007, *p* = 0.931]. Significant interactions existed for *Gain* between frequency and time [*F*_(1, 65)_ = 7.577, *p* = 0.008, partial eta^2^ = 0.104] and between group and time [*F*_(1, 65)_ = 5.898, *p* = 0.018, partial eta^2^ = 0.083], and for *Phase* between frequency and time [*F*_(1, 65)_ = 3.982, *p* = 0.049, partial eta^2^ = 0.059]. Subsequent analysis of simple effects revealed a 12.4% increase in 0.10 Hz *Gain* (95%CI = +0.096–0.238%MCAv/%MAP, *p* < 0.001) and a 9.0% decrease in 0.10 Hz *Phase* (95%CI = −0.005 to −0.096 rads, *p* = 0.027) from pre- to post-season in contact sport athletes (Figure 2), suggestive of autonomic dysfunction. No changes were observed at 0.10 Hz in control athletes (Figure 2) for *Gain* (95%CI = −0.079–0.115%/%, *p* = 0.696) or *Phase* (95%CI = −0.103–0.029 rads, *p* = 0.247). No significant changes were observed for 0.05 Hz *Gain* or *Phase* in either group (*p* > 0.05).

### Relation to head impacts

Comparing high-vs.-low quartiles of total number of hits experienced by the contact sport athletes revealed significant differences in Δ*Phase* at 0.10 Hz–players experiencing fewer hits exhibited a mean ± SD increase in *Phase* of 0.092 ± 0.108 rads from pre- to post-season, whereas those experiencing more hits exhibited a decrease in *Phase* (i.e. impairment) of 0.070 ± 0.067 rads [*t*_(12)_ = 2.366, *p* = 0.036]. Similarly, Δ*Phase* at 0.10 Hz differed between high-vs.-low quartiles of *cPLA*, wherein players exposed to lower cumulative linear acceleration exhibited a *Phase* increase of 0.092 ± 0.108 rads, while those in the highest *cPLA* quartile exhibited a *Phase* decrease of 0.043 ± 0.107 rads [*t*_(12)_ = 3.381, *p* = 0.005].

### Relation to symptom scores

For the contact sport athletes, significant Spearman's correlations were observed between Δ*Phase* at 0.10 Hz and symptom scores for “pressure in the head” (rho = −0.344, *p* = 0.043), and between Δ*Gain* at 0.10 Hz and headache scores (rho = −0.512, *p* = 0.002). No significant correlations were observed between change in performance on a cognitive screening test (SAC) and ΔPhase (*r* = −0.101, *p* = 0.562) or ΔGain (*r* = 0.17, *p* = 0.328).

## Discussion

In the current report, we present transcranial Doppler ultrasound data showing impairments in both the *Gain* and *Phase* of the cerebral pressure-flow relationship in contact sport athletes who did not sustain a concussion during the season, but not in non-contact sport athletes. The observed deficits suggest a delayed change in vascular resistance in response to BP oscillations associated with a greater magnitude change in CBF. The data also demonstrate greater deficits in dCA in athletes exposed to a higher number of hits and higher *cPLA* compared to those exposed to lower head impact levels based on data recorded from the xPatch system, the limitations of which are emphasized below. Although the permanence of the observed dCA impairments has not been established, the current data suggest the potential for a season of subconcussive head impacts to impair dynamic cerebral autoregulation.

A number of studies have reported minimal or no detrimental effects of subconcussive head impact, particularly on clinical measures of behavior/neuropsychological function [reviewed in (31)]. Despite exposure to over 1,000 impacts in a collegiate football season, scores on common assessments of concussion were not meaningfully impaired, although a higher number of years of collegiate playing experience was associated with worse Sensory Organization Test performance (32). Thus, findings to date suggest neuropsychological function is relatively unaffected by repetitive subconcussive head impacts and/or that subconcussive sequelae may require more time to develop than a single season (31).

By contrast, evidence suggests repetitive subconcussive head impacts, in the absence of symptoms, are associated with disruptions in various aspects of brain structure and function, including white matter microstructure (6), cerebral metabolism (15), cortical activation patterns (10), vestibular function (33), and functional connectivity (9). While these studies provide important insight into this issue, it is important to consider potential shortcomings in this body of research. First, many studies which have undertaken the effort to relate head impact exposure to changes in brain structure or function have either not directly quantified head impact exposure or used impact monitoring systems whose accuracy has been questioned (11–14) thus potentially limiting any interpretations that could be made. Second, some of these studies have not included an appropriate non-contact sport control group to disentangle the effects of a season of competitive sports participation in general from the potentially more specific effects of exposure to head impacts induced by participating in a contact sport in particular. With these limitations in mind, the current results demonstrate impairments in physiological function, including the integrity of CBF control, following one season of play. Interestingly, increases in dCA gain over the course of the season were related to increased headache scores, while decreases in dCA phase were related to increased reporting of pressure-in-the-head (34). Finally, the dCA changes observed were unrelated to cognitive performance as assessed by the SAC consistent with previous reports of minimal effects of repetitive subconcussive head impacts on neuropsychological performance (31).

Although acute and chronic disruptions in CBF have been well-documented following concussions (35), our understanding of the association between subconcussive head impacts and cerebral perfusion is relatively modest. Svaldi and colleagues (7) demonstrated impairments in cerebrovascular reactivity in collegiate football players following the onset of the season. While the subconcussive nature of head impacts in professional boxing is debatable, Bailey and colleagues (22) reported impairments in CO_2_ reactivity related to the volume and intensity of sparring but not the frequency of knock-outs suffered or number of rounds fought. Considered alongside the current data these findings are concerning, as the underlying mechanisms governing CO_2_ reactivity and dCA are thought to represent distinct processes (36), suggesting repetitive subconcussive head impacts are associated with deficits to multiple aspects of cerebrovascular control.

An impaired dCA response at 0.10 Hz implies dysregulation in the autonomic control of the cerebrovasculature (27). Increasingly, the rich sympathetic innervation of the cerebrovascular tree is recognized to play an important role in the dynamic regulation of BP variability (37). Numerous lines of evidence have outlined the detrimental effects of concussion on autonomic function (20) including systematic alterations in dCA (21). In the current study, disruptions were observed in both the latency and magnitude of the dCA response following subconcussive head impacts, indicating even mild repetitive head impacts are associated with disruptions to CBF regulation during BP challenges. Additional research is warranted to further clarify the underlying mechanisms of cerebrovascular dysfunction, which may be influenced by age, sex, previous impact exposure, and baseline autonomic function.

Prior work has demonstrated the effects of repetitive blows to the head may be cumulative, with impact exposure correlating to pathologic alterations on functional MRI (38), white matter diffusion (39), and cerebral metabolism (40). For example, in collegiate football and ice hockey players, diffusion changes in multiple regions of white matter were associated with head impact exposure, including total number of hits and exposure to linear acceleration (39). Moreover, football players sustained a higher number and severity of head impacts prior to diagnosed concussions than on days without concussions, suggesting subconcussive impacts may incrementally alter cerebral susceptibility and thereby lower the threshold for injury (41). While it has been established there is no reliable biomechanical threshold for concussion incidence, the collective findings suggest subconcussive head impacts may influence injury risk. The current results associating change in dCA function with impact exposure should be considered preliminary, and further study is warranted to investigate potential long-term consequences of repetitive hits on cerebrovascular and neurologic function.

This study has a number of limitations that should be acknowledged. First, transcranial Doppler ultrasound measures the velocity of red blood cells, rather than CBF explicitly. For velocity to approximate flow in this scenario, the diameter of the insonated vessel must remain constant, which cannot be verified. However, debate continues over the importance of diameter changes in evaluating cerebral hemodynamics, particularly when P_ET_CO_2_ is held stable (42), as it was in this study. Second, while previous research has demonstrated head impact exposure is typically greater during games than practices (43), impact data were collected during games only and therefore underestimate absolute exposure levels. Were it available, undocumented impacts during practices may have altered the populations of high vs. low quartile groups; this seems unlikely however, as impact exposure has been related more closely to player characteristics [e.g., aggression (44), BMI (43), position (45, 46)], which are unlikely to change meaningfully between game and practice settings. Furthermore, the skin-worn impact sensors used in this study have been shown to exhibit appreciable *in-vivo* overestimation error due partly to non-rigid skull coupling among other factors (11, 47). Moreover, when tested in a biofidelic context, the X2 system has a number of other shortcomings which limit the extent to which the head impacts it records are a robust and accurate representation of the head impacts that actually occurred. Namely, proprietary internal ‘de-clacking’ algorithms—designed to reduce erroneous impact recordings—create a high false-negative rate, wherein a substantial portion of true simulated impacts were deemed “invalid” by the system (11). In addition, some impacts may be missed entirely (47). Furthermore, inconsistencies were noted based on which surrogate was used, and which side of the surrogate was equipped with the patch (11). We attempted to limit the influence of the latter factor by ensuring sensors were always worn on the participants' right, however the relative influence of impact location relative to device location cannot be assessed. How these numerous limitations may have affected the distribution of cumulative loading estimated in the current study cannot be discerened. Thus, our analysis was restricted to high vs. low quartiles of head impact exposure, but may nevertheless be of limited utility. Third, all of our participants were male and, given the known differences in both cerebral autoregulation (48) and responses to repetitive subconcussive head impacts (49) between the sexes, it is unlikely the results would be the same in female participants. Nevertheless, despite these limitations and toward the primary objective of this study, significant dCA impairments were observed in contact sport athletes but not in non-contact control athletes, thus emphasizing the need for in-depth prospective investigations into the effects of subconcussive head impacts on CBF control mechanisms.

## Conclusion

There is growing concern that even low-magnitude, subconcussive head impacts can cause lasting neurological injury. Whereas, behavioral changes in response to subconcussive head impacts have been difficult to identify, this study suggests the cerebral autoregulatory system is vulnerable to repetitive head impacts. Our data provide evidence of cumulative impairment in the dCA response of contact sport athletes associated with exposure to repetitive head impacts. Importantly, non-contact athletes exhibited no such changes in dCA integrity. Future prospective cohort studies in a larger number of subjects are warranted to investigate the clinical relevance of dCA changes induced by subconcussive head impacts toward injury susceptibility and long-term outcomes.

## Author contributions

AW and PvD designed the study. AW, JS, and KB performed data collection, and AW performed the analyses, interpreted the data, and wrote the manuscript. All authors had full access to the data, and helped critically revise the manuscript before reviewing and approving the final version.

### Conflict of interest statement

The authors declare that the research was conducted in the absence of any commercial or financial relationships that could be construed as a potential conflict of interest.
